# Effect of the Extruded Pea Hulls on Physicochemical and Sensory Properties of Wheat Bread

**DOI:** 10.3390/foods13243985

**Published:** 2024-12-10

**Authors:** Dace Klava, Ruta Galoburda, Ilze Gramatina, Evita Straumite, Agris Staugis, Sanita Reidzane

**Affiliations:** 1 Food Institute, Latvia University of Life Sciences and Technologies, Riga Street 22, LV-3004 Jelgava, Latvia; dace.klava@lbtu.lv (D.K.); evita.straumite@lbtu.lv (E.S.); 2 ASNS Ingredient Ltd., Lietuvas Str. 16A, LV-3023 Eleja Parish, Jelgava Region, Latvia

**Keywords:** pea by-product, dietary fiber, wheat bread, dough rheological properties

## Abstract

Incorporating extruded pea hulls (EPH) into wheat bread increases its nutritional value by increasing dietary fiber content, which in turn influences the physicochemical properties and sensory attributes of bread. This study aimed to assess the effect of varying EPH levels on the rheological properties of wheat dough, as well as on the physical and sensory attributes of wheat bread, providing insight into the optimal EPH inclusion level. Farinograph analysis indicated that the inclusion of extruded pea hulls progressively increased the water absorption capacity. At higher EPH replacement levels, bread exhibited decreased specific volume, increased hardness, reduced porosity, darker color, and pronounced sensory attributes of pea aroma and pea taste. Bread with 5–15% EPH retained physical qualities comparable to bread without EPH, with 5% EPH replacement particularly improving specific volume, porosity, and texture. However, 20–30% EPH significantly impaired bread quality, resulting in denser crumb, darker crumb color, and intensified pea aroma. These findings suggest that moderate EPH inclusion (up to 15%) is optimal for enhancing fiber content without compromising bread quality, while higher levels (20% and more) may negatively affect both physical and sensory attributes.

## 1. Introduction

Peas (*Pisum sativum* L.) are an environmentally sustainable crop and a valuable source of plant-based protein [1], whose popularity has grown due to shifting dietary preferences. Pea seeds are processed to extract protein and starch, producing concentrates and isolates, with the hull (seed coat) being generated as a by-product [2]. Pea hulls are recognized for their potential to improve dietary fiber content in various food products [3]. The primary component of pea hull fiber is insoluble fiber, followed by smaller amounts of starch and soluble fiber [4]. This insoluble fiber mainly consists of cellulose, hemicellulose, lignin, and pectin, which provide structural rigidity and beneficial functional properties such as water-binding capacity, which increases dough and bread yield [5,6]. Dietary fibers reduce the staling process of wheat bread, thereby extending its shelf life. Thus, pea hulls could serve as a novel raw material for bread production. In addition to dietary fiber, pea hulls contain minor amounts of starch, protein, minerals, and antioxidants, making them a valuable additive for improving the nutritional profile of fiber-enriched products [2,7].

Several studies have demonstrated that fiber from various sources, such as wheat bran, vegetable, and fruit fiber, can be incorporated into bread by replacing refined wheat flour [8,9,10,11], which is often deficient in vital nutrients due to processing. The inclusion of fiber at levels ranging from 1% to 25% as a substitute for wheat flour has been extensively studied [10,11,12,13]. Among these, wheat bran remains the most commonly used fiber for producing fiber-rich bread, although food by-products from legumes are increasingly being explored as alternative sources of fiber [14].

The impact of fiber incorporation is typically assessed by evaluating dough rheology, loaf volume, crumb texture and quality, crust color, and flavor [15]. A growing trend in the modern market focuses on developing products that combine health benefits with appealing sensory attributes [8]. Paladugula et al. [16] found that up to 20% pea flour could be incorporated into wheat bread without significantly affecting consumer acceptance.

Despite their nutritional advantages, the use of pea hulls in food production remains limited due to several sensory and techno-functional challenges, including pea aroma and taste, low solubility, poor water binding capacity, large particle size, and high resistance to size reduction. These drawbacks have restricted the broader application of pea hulls in the food industry [5,15]. The functionality of fibers, crucial for their use, depends on their physicochemical properties and the processing conditions of the by-products [15].

Extrusion technology offers a promising approach to address these limitations by enhancing the sensory appeal of fiber-rich ingredients and modifying their physicochemical properties [13]. For fiber-rich products, extrusion can increase solubility, modify fiber functionality, and facilitate smoother incorporation into various food matrices. During extrusion, the breakdown of cell walls in the hull increases fiber solubility and alters the ratio of soluble to insoluble fiber [17,18]. These structural modifications improve functional properties such as water absorption and viscosity [18].

Insoluble fiber, like wheat bran, is known to weaken gluten networks, changing the elasticity and gas retention of dough and often resulting in reduced loaf volume and denser bread texture. However, extruded wheat bran has demonstrated improved effects on dough elasticity and stability, particularly in low-gluten formulations [13]. This suggests that extrusion could also improve the functional compatibility of pea hulls with wheat dough. Wheat bread is a traditional staple food in many countries, and often it is made from white wheat flour, which is rich in carbohydrates but lacks bioactive compounds and dietary fiber, which possess many health benefits, such as reducing the risk of coronary heart diseases. It is excellent for weight management and reduces the risk of diabetes [19]. Thus, to improve the health of the population, bread could be a promising vehicle for required ingredient delivery.

Consequently, it is essential to investigate the incorporation of extruded pea hulls into wheat dough, with an emphasis on assessing technological properties such as water absorption, dough development time, and dough stability. By evaluating the effects of varying levels of extruded pea hulls on the physicochemical properties of wheat bread, this study aimed to provide insights into the optimal incorporation of extruded pea hulls in bread formulations.

## 2. Materials and Methods

The analysis was carried out in the laboratories of the Latvia University of Life Sciences and Technologies.

### 2.1. Wheat Flour and Extruded Pea Hull Characteristics

**Figure 1:**
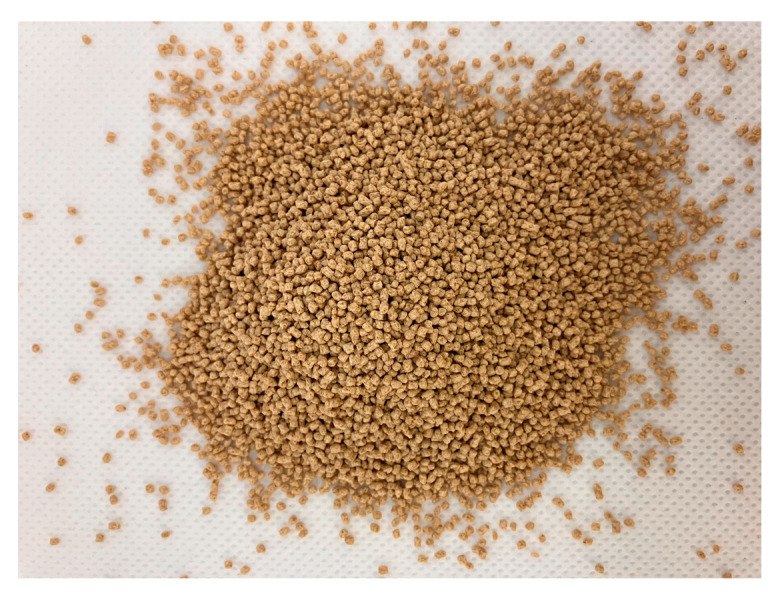
Extruded pea hull.

The commercial wheat flour (*Triticum aestivum* L.), Type 550 (AS Dobeles dzirnavnieks, Dobele, Latvia), and extruded pea hulls (ASNS Ingredient, Jelgava, Latvia) made from yellow peas (*Pisum sativum* L.) were used in the study. Physico-chemical properties of extruded pea hulls (EPH) obtained from the producer are dry matter 91%, ash 7.0%, particle size 1.5 mm, water binding capacity 1:5, and neutral taste (Figure 1).

### 2.2. Preparation of Composite Wheat and EPH Flour

In this study, six composite flours were prepared alongside a control sample containing 100% wheat flour (WB-0). In the other samples, wheat flour was progressively replaced with EPH at levels of 5% (WB-5), 10% (WB-10), 15% (WB-15), 20% (WB-20), 25% (WB-25), and 30% (WB-30). The composite flours were prepared by mixing EPH and wheat flour in a dough mixer (Kenwood, Hampshire, UK) for 5 min. These flour blends were then used both to analyze the rheological properties of dough and to prepare bread.

### 2.3. Analysis of the Rheological Properties Using Farinograph

Farinograph measurements were performed according to the standard method AACC 54-21.02 [20] using Farinograph-AT (Brabender GmbH & Co. KG, Duisburg, Germany). Water absorption (measured at 500 BU), dough development time, and dough stability were evaluated in duplicate for each sample.

### 2.4. EPH Containing Wheat Bread Preparation

The following ingredients were added based on the weight of composite flour: 1.5% fine salt (Artemsil, Soledar, Ukraine), 3.0% white sugar beet sugar (Nordic Sugar AS, Kedainiai, Lithuania), 3.0% fresh compressed yeast (*Saccharomyces cerevisiae* L.) (Lesaffre Polska SA, Wolczyn, Poland), and 3.0% refined sunflower oil (Cargill Oil Packers BVBA, Izegem, Belgium). Water at 28 °C was added as determined by farinograph measurement. The dough was kneaded using a Kenwood mixer (Hampshire, UK) and proofed in a Sveba Dahlen proofer (Fristad, Sweden) at 25 °C for 40 min. After proofing, the dough was divided into 280 ± 20 g pieces, placed in molds, and proofed again at 35 °C for 45 min. Baking of all samples was conducted at 200 ± 5 °C, with 4 s of steam at the beginning of baking, for 17 min in an S400 Mini Rack oven (Sveba Dahlen AB, Fristad, Sweden), after which the loaves were cooled at room temperature. Two independent bread production batches (experimental replicates) were conducted, each yielding four loaves per formulation.

After 4 h, the physicochemical properties of the EPH-containing wheat bread were determined. One loaf of each sample was placed in a polypropylene bag (thickness 25 μm, size 200 × 350 mm, Ltd. Velplev, Riga, Latvia), sealed with a clip, stored at room temperature 22 ± 1 °C for 3 days, and then analyzed. The experiment was performed in duplicate.

### 2.5. Determination of Physicochemical Properties of EPH Containing Wheat Bread

The bread moisture was measured according to AACC 44-19 [21]. Rapeseed displacement method AACC 10-05.01 [22] was used for bread volume determination. Specific volume was calculated as the ratio of bread volume to weight. Bread porosity was measured using the Zhuravlev device (Biomer Ltd., Krasnoobsk, Russia) according to the method described by Cizeikiene et al. [23]. The bread moisture, specific volume, and porosity were analyzed for each batch in duplicate.

Bread texture evaluation was performed using a TA.HD.Plus texture analyzer (Stable Microsystems, Godalming, UK) according to AACC 74-09 [24]. The trigger force was set at 0.049 N. Two slices of mechanically sliced bread (each 7 mm thick) were compressed with a 25 mm aluminum cylindrical probe at 1.7 mm s^−1^ speed, a distance of 5 mm. The hardness is a peak force of the compression curve, representing the crumb’s resistance to deformation. Texture measurements were repeated on twenty slices per bread sample. Hardness was measured on the baking day after cooling and on the 3rd day of storage.

### 2.6. Assessment of Bread Crumb Color

The color of wheat bread crumb with added EPH was assessed using a ColorFlex instrument (Hunter LAB, Reston, VA, USA) based on the CIE L*a*b* color system with a D65/10 illuminant. The instrument was calibrated against black and white tiles before measurement. The L* value indicates the brightness of the bread (with 100 representing white and 0 representing black), while a* represents redness and b* indicates yellowness. For each measurement, three randomly selected bread slices were stacked, and a single measurement in the central part of the slice was performed. Color measurements were repeated on ten slices per bread sample.

### 2.7. Total Dietary Fiber and Insoluble Dietary Fiber Evaluation

Total dietary fiber (TDF) was determined using the enzymatic-gravimetric method in three replications according to the standard method AOAC 985.29 [25]. Insoluble dietary fiber (IDF) was analyzed according to the standard method AOAC 991.42 [26]. A total dietary fiber kit (Megazyme, Wicklow, Ireland) was used. Soluble dietary fiber was calculated as TDF minus IDF.

### 2.8. Sensory Evaluation of Bread

In the evaluation, 36 panelists aged between 23 and 73 (66% women), who consume wheat bread regularly, participated. The panelists were recruited from the students and staff of the Food Institute and had received basic training in sensory evaluation. Each panelist was informed about the objectives of this study and provided informed consent, agreeing to participate voluntarily. Their responses were kept confidential, and they were given the option to withdraw from the evaluation at any time without providing a reason. All participants were in good health and reported no allergies or intolerance to wheat, ensuring their safety during the study.

The sensory evaluation was performed in a laboratory at the Food Institute at Latvia University of Life Sciences that complies with the ISO 8589:2007 standard [27] in one session. Each panelist received a slice of bread from each sample, with all samples coded with three-digit numbers and presented in the randomized order. Warm black tea was served as a palate cleanser between samples.

To assess the effects of EPH on the sensory properties of bread—such as crumb color, porosity, pea aroma, pea taste, and grainy aftertaste—a unipolar 12 cm line scale (ISO 4121) was used [28]. The scale ranged from 0–12, where crumb color was scored 0 for light/white; 12 for gray/dark gray, and porosity was scored 0 for small, even pores, with unpronounced porosity and 12 for large, uneven pores, very pronounced porosity.

The evaluation sheets, data collection, and data processing were conducted using FIZZ Acquisition 2.51 software (Biosystems, Couterno, France).

### 2.9. Statistical Analysis

Statistically significant differences (*p* < 0.05) between samples were assessed using pairwise comparisons with Tukey’s honest significant difference test. Pearson correlation was performed to determine the relationship between variables. Statistical analysis was carried out using open-source Python 3.10.12.

## 3. Results

### 3.1. Technological Characteristics of Wheat Dough Containing EPH

Water absorption increased with the amount of added EPH (Table 1), following a trend similar to that observed with the addition of extruded wheat bran to wheat dough [13,29]. Substituting wheat flour with 5% EPH extended the development time to 14.3 min. However, replacing a larger proportion of flour with EPH reduced the development time to 9.6 min to achieve the desired consistency. These observations align with those of Gomez et al. [29], who reported comparable effects when incorporating extruded wheat bran into a dough.

The enhanced water absorption in dough containing EPH can be attributed to structural changes in the fibers induced by extrusion. In pea hull fibers, cellulose and hemicellulose form a compact, well-ordered network within the pectin matrix, which limits interaction with water [5]. This tightly cross-linked network also limits the solubility of soluble fibers (pectin). High-pressure extrusion disrupts this rigid matrix, increasing its water-binding capacity. Similarly, Li et al. [13] reported that increased water absorption in wheat dough with extruded bran is linked to the presence of hydrophilic groups in soluble fiber, along with starch damage. They also noted that the development time of dough with added extruded bran varies depending on the gluten content [13]. Fiber incorporation may interfere with the gluten network, creating competition for water and impacting interactions between gluten and other dough components.

### 3.2. Dietary Fiber Content in EPH and Wheat Bread Containing EPH

The total dietary fiber content in EPH was found to be 58.94%, with insoluble fiber constituting 53.23% of the dry weight. This results in a very high proportion of 91.3% of insoluble dietary fiber. In a study by Ralet et al. [17], the solubilization of total dietary fiber from extruded pea hulls was reported to range from 1% to 7%, with the proportion of soluble fiber increasing depending on extrusion parameters such as temperature, screw speed, and specific mechanical energy. In contrast, Li et al. [13] reported that extruded wheat bran contains 7.65% soluble dietary fiber and 33.31% insoluble dietary fiber.

Analysis of the dietary fiber in wheat bread containing EPH revealed a clear trend: increasing EPH inclusion in wheat flour from 5% to 30% raised the total dietary fiber content in bread from 13.3% to 28.7% (dry weight) (Table 2).

The insoluble dietary fiber content ranged from 88–99% of the total dietary fiber, indicating a low presence of soluble dietary fiber in EPH-containing wheat bread. Both refined wheat flour and EPH contain minimal amounts of soluble fiber, leading to a low overall soluble fiber content in the bread [12]. Incorporation of 5% to 30% EPH in wheat flour qualifies the bread as a “high fiber” product according to nutrition claims, which require at least 6 g 100 g^−1^ [30]. Scientific articles indicate the beneficial effects of insoluble fiber from legumes on the digestive tract microbiota [6,31]. Wang et al. [32] indicate the development of beneficial bacteria (*Lactobacillus*) and the reduction of undesirable Bacteroides in the digestive tract when consuming products with IDF from legumes. Therefore, future studies should be conducted on the composition of IDF and its effects on the microbiota of human health.

### 3.3. Physicochemical Characteristics of Wheat Bread with EPH Inclusion

**Figure 2:**
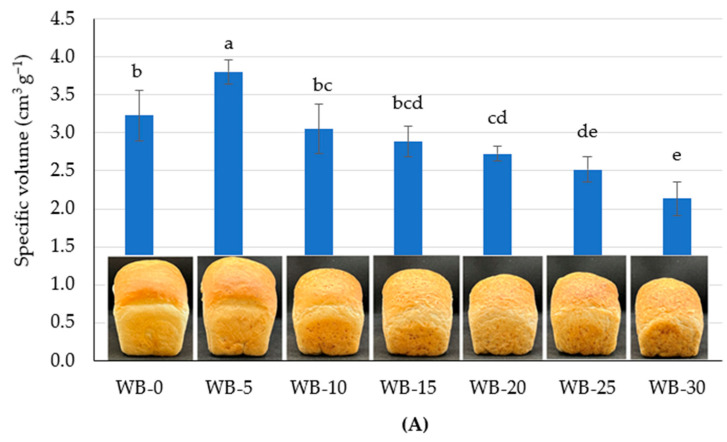

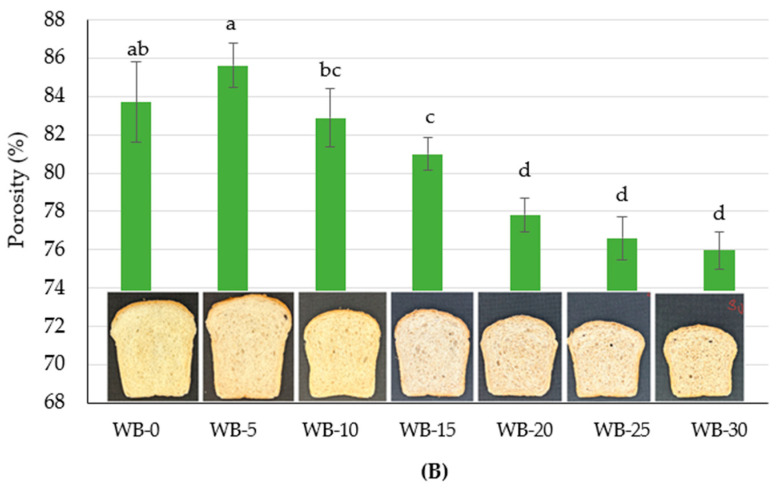
Specific volume (**A**) and porosity (**B**) of EPH-containing wheat bread (replacement level of wheat flour with EPH: 0, 5, 10, 15, 20, 25, 30%). Different letters a–e indicate significant differences between the samples (*p* ≤ 0.05).

The moisture content of analyzed bread samples was 43.3 ± 0.9% to 46.4 ± 1.3%. Figure 2A,B illustrates the effect of replacing wheat flour with extruded pea hulls on the specific volume and porosity of bread. As the EPH content in dough increased, the specific volume of wheat bread decreased from 3.2 ± 0.3 to 2.1 ± 0.2 cm^3^ g^−1^, while the sample WH-5 differed with the highest value of 3.8 ± 0.1 cm^3^ g^−1^. Bread porosity ranged from 76 ± 1% to 86 ± 1%, with sample WB-5 showing the highest result. No significant differences were observed in specific volume between control wheat bread (without EPH) and samples with 10% (WB-10) and 15% (WB-15). Similarly, replacing wheat flour with 20, 25, and 30% EPH did not result in significant changes in specific volume and porosity.

The reduction in bread loaf volume with increased dietary fiber content has been noted in other studies, which attribute this to the weakening gluten film, impairing carbon dioxide retention in dough [8]. The effect may also be due to lower substrate availability for yeast, leading to reduced gas formation.

This suggests that substituting 5%, 10%, and 15% of wheat flour with EPH did not have a negative impact on bread’s specific volume and porosity. A significant increase in specific volume was observed when 5% of flour was replaced with EPH. A similar trend was observed in bread containing 2.5% small-grained pea hulls, where this addition did not reduce bread volume [12]. However, when adding 5–10% small-grained pea hulls, Kasprzak et al. [12] similarly observed a reduction in specific volume and porosity, with a 5% addition resulting in lower specific volume wheat bread (2.4 cm^3^ g^−1^) and reduced bread porosity (55.6%). In contrast, adding 5% psyllium husk made the crumb of wheat bread softer and even significantly improved the crumb texture in bread with added coarse bran. This effect is due to the higher soluble fiber content in psyllium, which forms a gel. According to Li et al. [17], the addition of extruded wheat bran has also been shown to create a more polymerized and stable gluten network.

**Figure 3:**
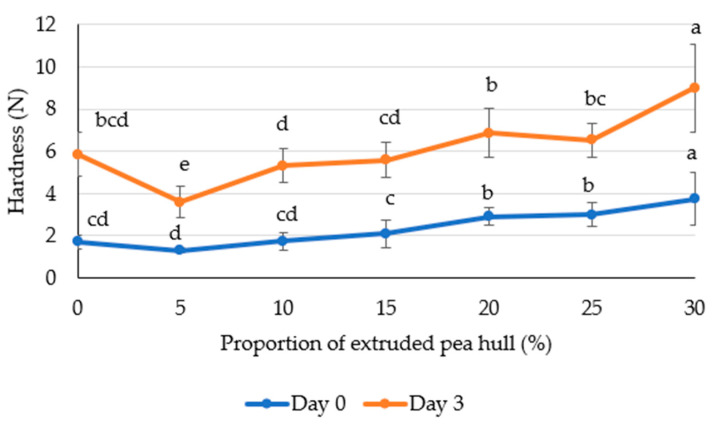
The hardness of wheat bread with EPH inclusion. Different letters (a–e) indicate significant differences (*p* < 0.05) between the samples measured on the same day.

At higher levels of EPH (20–30% flour replacement), a decrease in specific volume and porosity was observed. This may be due to the increased water-holding capacity of EPH [33], leading to competition between the added fibers and wheat gluten for water, which in turn affects gluten functionality [11]. Consequently, bread with higher EPH content was harder (Figure 3).

Wheat bread with a low replacement level of EPH at 5% was softer (1.3 ± 0.1 N) compared to the control (1.7 ± 0.3 N), while a 10% substitution did not significantly affect the texture (1.7 ± 0.4 N). The highest hardness of 3.7 ± 1.2 N was observed at a 30% replacement level. Ouyang et al. [34] suggested that a decrease in hardness may result from fiber adhering to gluten, which strengthens the gluten network and enhances the dough’s gas retention capacity. Gomez et al. [10] found that including extruded pea hulls significantly increased the hardness of wheat bread crumb at 20 g 100 g^−1^ compared to the control sample without added fiber.

The quality of bread during storage is determined by the mobility of water within the structural elements of the bread and the structure formed by starch, fiber, and proteins. As Li et al. [35] revealed, enzymatically extruded corn soluble fiber alters the starch gel structure by modifying the bonds between amylose and amylopectin, making them less rigid. As a result, bread with a 10% addition of such fiber exhibited reduced hardness during storage. In the EPH during pea hull extrusion, possibly cellulose derivatives are formed, which may also modify the structure of bread, potentially influencing its hardness during storage [36]. Therefore, analyses were conducted on samples after three days of storage.

Bread hardness increased after three days of storage to 5.8 ± 1.0 N for WB-0. Hardness for WB-30 was 9.0 ± 2.1 N. with the changes observed for bread containing EPH being smaller than those in bread without EPH. Specifically, hardness increased by approximately 130% in bread with 20–30% EPH, around 180% in samples with 5–15% EPH, and up 240% in bread without EPH. This trend may be attributed to the amount of water absorption capacity of fiber. During storage, moisture content in the bread crumb decreases due to the macroscopic migration of water, increasing hardness. Pan et al. [37] observed that adding 6% wheat bran reduced moisture loss in wheat bread crumb, attributed to its higher water absorption and water-holding capacity due to the presence of cellulose, hemicellulose, and pentosans.

**Figure 4:**
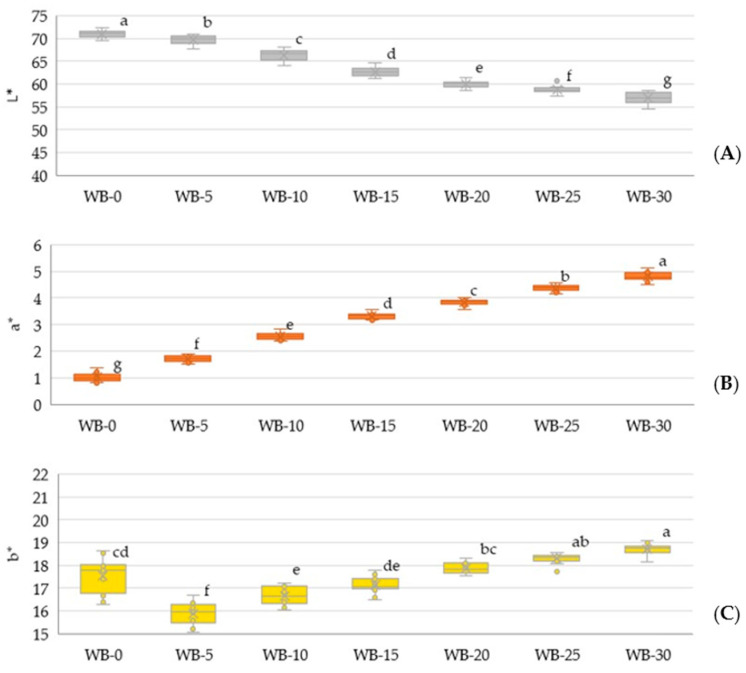
Color components of wheat bread with EPH inclusion: (**A**) L*—lightness; (**B**) a*—redness; (**C**) b*—yellowness. Different letters (a–g) indicate significant differences (*p* < 0.05) between the same color component of the samples.

Figure 4 presents the color parameters of wheat bread crumbs with EPH inclusion, measured using the Cie L*a*b* color system. As the fiber content increased, the color became darker and exhibited greater redness. This observation aligns with previous studies [14]. The darkness imparted to wheat bread may be attributed to the presence of natural pigments and minerals in fiber or reactions occurring during baking in the presence of higher water content. Additionally, reduced brightness may occur because fiber particles scatter light differently than starch.

### 3.4. Intensity of Bread Sensory Properties

Replacing wheat flour with extruded pea hulls (EPH) had a significant effect (*p* < 0.0001) on the intensity of bread sensory properties (Table 3). Replacing 20–30% of wheat flour with EPH significantly affected the crumb color, making it darker and grayer. This darker crumb color, combined with a stronger aroma, resulted in varying preferences among the panelists for bread samples with EPH substituting 20–30% of wheat flour. While some panelists appreciated the more pronounced color and taste, suggesting it could add value to the bread, others found the intense pea aroma and taste to be less appealing.

Pea-based ingredients often develop a distinct aroma and taste upon heating, which may not appeal to all consumers. However, a significantly pronounced pea taste was detected only in the WB-30 sample. In the bread sample containing a 70:30 ratio of wheat flour to EPH, a slightly floury, grainy aftertaste was also noted, likely due to the incomplete EPH dissolution in the dough.

Several studies also report that adding legumes such as peas and lentils to bread can significantly affect sensory properties including color, elasticity, aroma, and taste [9,38,39,40], supporting the findings of this study. All bread samples showed average porosity, with line scale values ranging from 4.38 (WB-30) to 6.44 (WB-20).

### 3.5. Correlations Between Physical, Chemical, and Sensory Properties of Bread

**Figure 5:**
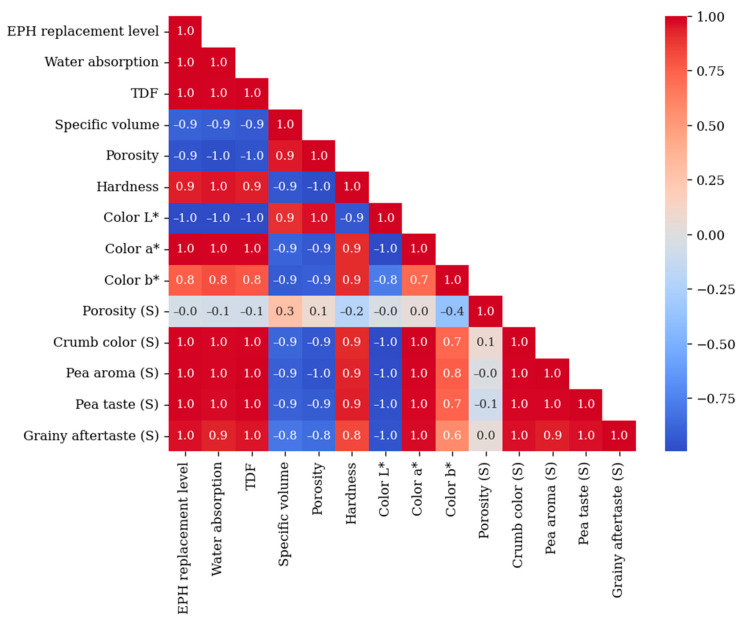
Correlation between physical, chemical and sensory attributes of a wheat bread containing EPH. TDF—total dietary fiber, (S)—sensory attributes.

The relationships between physical, chemical, and sensory attributes of wheat bread containing EPH revealed a high positive correlation between the EPH replacement level and water absorption, total dietary fiber content, and sensory properties such as crumb color, pea aroma, pea taste, and grainy aftertaste (Figure 5). Notably, pea aroma, pea taste, and grainy aftertaste are closely interconnected—a strong pea aroma is often accompanied by a stronger pea taste and a more pronounced grainy aftertaste.

Conversely, the EPH replacement level showed a negative correlation with specific volume, porosity, and color (L* value). As the replacement level increased, the bread volume decreased, the color became darker, and the texture became harder. Sensory porosity exhibited a weak negative correlation with hardness, color, and pea taste. Additionally, color properties were strongly associated with both physical and sensory characteristics, suggesting that color may serve as a key indicator of perceived sensory quality.

## 4. Conclusions

Extruded pea hulls (EPH) offer a valuable source of insoluble fiber and are suitable ingredients for producing high-fiber bread. The impact of EPH on bread properties varied with the replacement level. Higher EPH levels increased the dough’s water absorption and crumb hardness while reducing the bread’s specific volume and porosity. Hardness measurements after 3 days of storage revealed a significant overall increase, but the rise was significantly lower in the bread containing 5–15% EPH. Additionally, they intensified sensory attributes, particularly enhancing pea aroma and pea taste. Sensory evaluation showed that the pronounced aroma and taste associated with 20–30% EPH inclusion may be perceived either as a positive enhancement or an undesirable feature, depending on consumer preference. These findings suggest that EPH can be effectively used in moderate amounts (up to 15%) to enhance fiber content without adversely affecting bread quality. Notably, the inclusion of 5% EPH enhanced specific volume, porosity, and texture.

This study explored the application of extruded pea hull in refined wheat flour bread to enhance fiber content and improve the dough’s water absorption capacity. Substituting wheat flour at varying levels can be optimized to achieve different outcomes: at lower substitution levels, the bread’s physical properties remain largely unaffected, while higher substitution levels allow consumers to perceive the product as a specific high-fiber content option.

## Figures and Tables

**Table 1:** Technological properties of EPH-containing wheat dough.

EPH Replacement Level (%)	Water Absorption (%)	Dough Development Time (min)	Dough Stability Time (min)
0	60.2 ± 0.1 ^f^	1.4 ± 0.1 ^d^	8.5 ± 0.1 ^a^
5	61.3 ± 0.8 ^f^	13.8 ± 0.6 ^a^	8.2 ± 0.1 ^a^
10	63.9 ± 0.6 ^e^	12.1 ± 0.2 ^b^	8.3 ± 0.9 ^a^
15	67.4 ± 0.2 ^d^	10.9 ± 0.7 ^bc^	4.2 ± 0.1 ^b^
20	70.4 ± 0.2 ^c^	10.4 ± 0.2 ^c^	3.4 ± 0.0 ^c^
25	73.4 ± 0.1 ^b^	9.6 ± 0.0 ^c^	2.6 ± 0.0 ^de^
30	75.4 ± 0.6 ^a^	9.6 ± 0.1 ^c^	2.5 ± 0.1 ^e^

Letters (a–f) indicate significant differences (*p* ≤ 0.05) between the samples in columns.

**Table 2:** Dietary fiber content in EPH-containing wheat bread.

Samples ^1^	Total Dietary Fiber (% dw)	Insoluble Dietary Fiber (% dw)	Soluble Dietary Fiber (% dw)	Total Dietary Fiber (100 g^−1^ product)
WB-0	11.05 ± 0.21 ^g^	10.98 ± 0.17 ^g^	0.10 ± 0.03 ^e^	4.19 ± 0.29 ^g^
WB-5	13.35 ± 0.21 ^f^	13.04 ± 0.18 ^f^	0.31 ± 0.01 ^d^	7.88 ± 0.13 ^f^
WB-10	18.38 ± 0.38 ^e^	18.04 ± 0.06 ^e^	0.42 ± 0.01 ^c^	10.41 ± 0.21 ^e^
WB-15	20.40 ± 0.31 ^d^	19.80 ± 0.09 ^d^	0.59 ± 0.04 ^b^	11.32 ± 0.17 ^d^
WB-20	22.88 ± 0.08 ^c^	22.66 ± 0.10 ^c^	0.27 ± 0.08 ^d^	12.40 ± 0.04 ^c^
WB-25	25.66 ± 0.17 ^b^	25.39 ± 0.23 ^b^	0.27 ± 0.05 ^d^	13.42 ± 0.09 ^b^
WB-30	28.66 ± 0.0.19 ^a^	27.90 ± 0.11 ^a^	0.70 ± 0.01 ^a^	15.19 ± 0.10 ^a^

^1^ The number in the sample name indicates the percentage of flour substituted. Letters (a–g) indicate significant differences (*p* ≤ 0.05) between the samples in columns.

**Table 3:** The intensity of sensory properties of analyzed bread samples.

Bread Samples	Sensory Properties				
	Crumb Color	Porosity	Pea Aroma	Pea Taste	Grainy Aftertaste
WB-0	1.63 ^f^	4.64 ^bc^	0.17 ^e^	0.14 ^d^	1.33 ^e^
WB-5	3.23 ^e^	5.69 ^ab^	0.77 ^d^	0.93 ^d^	2.41 ^d^
WB-10	5.09 ^d^	5.42 ^b^	2.74 ^c^	2.01 ^c^	2.92 ^cd^
WB-15	6.43 ^c^	5.75 ^ab^	2.79 ^c^	2.38 ^c^	3.49 ^bc^
WB-20	8.54 ^b^	6.44 ^a^	4.35 ^b^	3.36 ^b^	3.48 ^bc^
WB-25	8.84 ^b^	5.37 ^b^	5.12 ^ab^	3.64 ^b^	3.82 ^ab^
WB-30	10.20 ^a^	4.38 ^c^	5.90 ^a^	5.23 ^a^	4.54 ^a^
*p*-value	*p* < 0.00001	*p* = 0.0315	*p* < 0.0001	*p* < 0.0001	*p* < 0.001

Different letters (a–f) indicate significant differences between the samples in column.

## Data Availability

The original contributions presented in this study are included in the article material. Further inquiries can be directed to the corresponding author.
